# Health-related quality of life in populations with diabetes, prediabetes, and normal glycemic levels in Guangzhou, China: a cross-sectional study

**DOI:** 10.3389/fendo.2025.1518204

**Published:** 2025-05-23

**Authors:** Meichen Li, Na Liu, Guli Jiang, Hongli Zeng, Jianwen Guo, Darong Wu, Hui Zhou, Zehuai Wen, Li Zhou

**Affiliations:** ^1^ The Second Clinical Medical College of Guangzhou University of Chinese Medicine, Guangzhou, China; ^2^ Department of Health Management Center, Guangzhou 11th People’s Hospital, Guangzhou, China; ^3^ State Key Laboratory of Dampness Syndrome of Chinese Medicine, Second Affiliated Hospital of Guangzhou University of Chinese Medicine, Guangzhou, China; ^4^ Center for Clinical Research, Second Affiliated Hospital of Guangzhou University of Chinese Medicine, Guangzhou, China

**Keywords:** diabetes, prediabetes, health-related quality of life, EQ-5D, EQ visual analog

## Abstract

**Background:**

The association of prediabetes and diabetes on health-related quality of life (HRQoL) remains inconclusive in current epidemiological research. In this investigation, we administered 5-level EuroQoL-5 dimension version (EQ-5D-5L) to systematically assess HRQoL across glycemic strata (diabetes, prediabetes, and normal glycemic levels) in Guangzhou, and to offer baseline data that can be easily compared to other regions in China or across countries.

**Method:**

This investigation utilized baseline cross-sectional data extracted from a three-year prospective cohort study conducted at the Health Management Center of Guangzhou 11th People’s Hospital. Propensity score matching was implemented at a 1:1:4 ratio to balance participants across diabetes, prediabetes, and normal glycemic group. HRQoL outcomes, operationalized through EQ-Index and EQ visual analog scale (EQ-VAS) measurements, were compared across groups using one-way ANOVA or Wilcoxon rank-sum tests. Multivariate linear regression was constructed to adjust for potential confounders, followed by subgroup analyses stratified by sex, age categories, body mass index (BMI) classifications, and hypertension comorbidity status.

**Results:**

A total of 18,605 participants were included in the study. After propensity score matching, 533 participants allocated to the prediabetes group, 533 to the diabetes group, and 2064 to the normal glycemic group. Intergroup comparisons demonstrated significantly lower EQ-VAS scores in the diabetes group (79.11) compared to both prediabetes (80.67) and normal glycemic group (81.65). Similarly, the diabetes group exhibited the lowest EQ-Index scores (0.968) relative to prediabetes (0.972) and normal glycemic group (0.972). Multivariate linear regression adjusted with sex, age, BMI, etc. revealed a 2.139-point reduction in EQ-VAS scores for the diabetes group versus normal glycemic group (95% CI: -3.748, -0.530; *P*=0.009). Subgroup analyses identified particularly compromised HRQoL in diabetes and prediabetes populations among female participants, individuals with obesity, and those aged ≥60 years.

**Discussion:**

Prediabetes and diabetes mellitus are associated with diminished HRQoL compared to normal glycemic levels, with a more pronounced negative associations observed among female populations, older adults, and individuals with obesity. These findings emphasize the clinical necessity for implementing targeted interventions to optimize HRQoL outcomes in these high-risk subgroups, which aligns with the fundamental objectives of contemporary diabetes management frameworks.

**Clinical trial registration:**

ClinicalTrials.gov ID: NCT05315895.

## 1 Introduction

Type 2 diabetes mellitus (T2DM) represents a significant global public health challenge. According to World Health Organization projections, the global burden of T2DM is anticipated to rise to 700 million cases by 2045, with an estimated prevalence of 10.9% worldwide (1, 2). China poses a formidable public health challenge, with national surveillance data indicating that 15.5% of the adult population meets glycemic criteria for either prediabetes or diabetes (3–6). Diabetes-associated complications —such as neuropathy (prevalence range: 28-45%), retinopathy (19-28%), and cardiovascular disease (32-49%)—impose significant long-term morbidity burdens (7, 8). The cumulative association of these physical manifestations and associated psychological stressors significantly compromises patients’ health-related quality of life (HRQoL).

HRQoL measurement has emerged as a critical metric for evaluating diabetes management outcomes, integrating physical, psychological, and social functioning domains (9). Evidence from cross-European studies indicates that approximately 75% of individuals with T2DM exhibit clinically significant HRQoL impairment (10, 11). This epidemiological pattern has been corroborated in population-based research from Hong Kong (12), Shanghai (13), and Suzhou (14), confirming cross-cultural consistency in HRQoL impairment profiles associated with T2DM. However, the association of the prediabetes stage on HRQoL remains insufficiently characterized, despite its role as a precursor to clinical diabetes. Leal et al. (15) reported that both prediabetes and diabetes were associated with significantly lower HRQoL compared to normoglycemia in a Danish research. In contrast, Makrilakis et al. (16) found no significant difference in HRQoL between Greek prediabetes and healthy controls, though diabetes scored lower. Notably, a paradoxical observation emerged from Sichuan, China, where prediabetes demonstrated higher HRQoL scores than both normoglycemic and diabetes (17). These contradictory findings underscore critical knowledge gaps regarding the heterogeneous effects of different glycemic states and different geographically distinct populations on well-being (18, 19). To reconcile these discrepancies, large-scale studies with rigorous adjustment for confounders and population-specific sociocultural contexts are needed. These findings underscore the need to conduct studies within diverse Chinese subpopulations(e.g., rural/urban populations and geographic regions) to establish baseline data enabling systematic comparisons across Chinese regions and internationally.

The EuroQoL-5 dimension version, a generic preference-based instrument for quality of life assessment, quantifies five core dimensions: mobility, self-care, usual activities, pain/discomfort, and anxiety/depression (20–22). Developed as an enhanced iteration of the original 3-level EuroQoL-5 dimension version (EQ-5D-3L), the 5-level version (EQ-5D-5L) was designed to address ceiling effects and enhance measurement precision through expanded response categories (23). Extensive validation studies have established its construct validity, responsiveness to clinical changes, and cross-population comparability in diverse healthcare contexts (23). The Chinese version of the EQ-5D-5L has undergone comprehensive cross-cultural adaptation, demonstrating acceptable internal consistency (Cronbach’s α = 0.624) for epidemiological research (24).

In this study, we conducted the EQ-5D-5L instrument to assess HRQoL among individuals categorized into three glycemic subgroups (diabetes, prediabetes, and normoglycemia) in Southern China. By generating region-specific HRQoL reference profiles aligned with WHO measurement frameworks, this work establish reference data for cross-regional and cross-national comparative analyses.

## 2 Methods and analysis

### 2.1 Ethical standard and study registration

This study protocol has been approved by the Ethics Committee of the Guangdong Provincial Hospital of Chinese Medicine (Ref No. BE2021-247-04) and the Ethics Committee of the Guangzhou 11th People’s Hospital (Ref. No. K2022-03). The study was registered with the Clinical Trial Registry (ClinicalTrials.gov, ID: NCT05315895) on November 14, 2022.

### 2.2 Study design

This cross-sectional study was the first survey from a population-based prospective cohort study conducted by the Guangdong Provincial Hospital of Chinese Medicine in collaboration with the Guangzhou 11th People’s Hospital.

### 2.3 Study population

From May 1, 2022, to December 31, 2023, a total of 26,472 subject were enrolled at the Health Management Center in Guangzhou 11th People’s Hospital. The study employed a dual-stage screening process encompassing (1) certified physicians and research personnel systematically performed standardized eligibility evaluations using predefined inclusion/exclusion criteria, followed by (2) face-to-face eligibility confirmation through structured clinical assessments, issuing formal written invitations to potential participants. This methodological framework was specifically designed to ensure study integrity and adherence to protocol-specified selection parameters. All participants with diabetes, prediabetes, or normal glycemic levels included in the study were diagnosed by medical professionals. All study subjects signed the informed consent form. The personal information of the participants will be strictly protected by some strict protective measures, including anonymization, authority, and using strong passwords for the data-accessing process.

### 2.4 Diagnostic criteria

The diagnosis for the diabetes group followed the ADA criteria (25), including hemoglobin A1C (HbA1C) ≥ 6.5%, fasting plasma glucose (FPG) ≥ 126 mg/dl (7.0 mmol/L), oral glucose tolerance test (OGTT) ≥ 200 mg/dl (11.1mmol/L), or random plasma glucose (PG) ≥ 200 mg/dl. The diagnosis for the prediabetes group was also performed with adherence to ADA guidelines (25) This process involved identifying individuals with FPG levels between 100–125 mg/dl (5.6-6.9 mmol/L), OGTT levels between 140–199 mg/dl (7.8-11.0 mmol/L), or an HbA1C level between 5.7%-6.4%. Conversely, the rest of the people were listed with normal glucose levels.

### 2.5 Inclusion and exclusion criteria

The eligible subjects met the following criteria: (1) age 18 years old or above; (2) completion of routine physical examination at the Guangzhou 11th People’s Hospital, Guangzhou, China. There were no gender restrictions for the participants. Exclusion criteria comprised: (a) severe cognitive impairment (medical record-documented diagnosis); (b) active psychiatric disorders; (c) communication barriers; (d) critical systemic illnesses requiring intensive care; (e) incomplete EQ-5D-5L assessments. All subjects had informed consent and signed an informed consent form.

### 2.6 Variables and measurements

Data were collected on-site through a preplanned data collection form (DCF), including electronic or paper questionnaires, an EQ-5D-5L instrument, and a physical examination. The questionnaires in DCF were validated by endocrinology experts and data manager, consisting of 10 items to measure the life behavior of individuals. It covered various aspects, including sociodemographic factors (education levels, marital status, annual income, and family history of diabetes), lifestyle habits (cigarette smoking and alcohol drinking), sleep patterns (bedtime, length of sleep), and hypertension comorbidity. The detailed classification was shown in **Table 1**. Face-to-face interviews between the investigator and the subjects collected demographic information. The medical staff at the physical examination center collected the laboratory data.

**Table 1:** General characteristics by the groups after PS matching.

Characteristic	Diabetes mellitus (n=533)	Prediabetes (n=533)	Normal glycemic (n=2,064)	*P*
	N (%)	N (%)	N (%)	
Sex				0.975
Male	421 (79.0)	420 (78.8)	1,635 (79.2)	
Female	112 (21.0)	113 (21.2)	429 (20.8)	
Age				0.149
≤60	357 (87.0)	325 (61.0)	1,385 (67.1)	
>60	176 (33.0)	208 (39.0)	679 (32.9)	
BMI				0.732
<24	175 (32.8)	187 (35.1)	673 (32.6)	
24-28	254 (47.7)	247 (46.3)	1,001 (48.5)	
>28	104 (19.5)	99 (18.6)	390 (18.9)	
Cigarette smoking				0.609
Non-smoker	346 (64.9)	369 (69.2)	1,414 (68.5)	
Former smoker	61 (11.4)	57 (10.7)	195 (9.4)	
≤20 year	48 (9.0)	40 (7.5)	172 (8.3)	
>20 year	78 (14.6)	67 (12.6)	283 (13.7)	
Alcohol drinking				0.001
Non-Alcohol	241 (45.2)	166 (31.1)	936 (45.3)	
Former drinker	21 (3.9)	19 (3.6)	57 (2.8)	
<5 times/week	260 (48.8)	340 (63.8)	1,051 (50.9)	
≥ 5 times/week	11 (2.1)	8 (1.5)	20 (1.0)	
Education levels				0.249
≤Bachelor degree	72 (13.5)	77 (14.4)	247 (12.0)	
>Bachelor degree	461 (86.5)	456 (85.6)	1,817 (88.0)	
Marital status				0.063
Unmarry	17 (3.2)	7 (1.3)	27 (1.3)	
Divorce	21 (3.9)	22 (4.1)	100 (4.8)	
Widow	12 (2.3)	18 (3.4)	44 (2.1)	
Marry	460 (86.3)	469 (88.0)	1,805 (87.5)	
Remarry	23 (4.3)	17 (3.2)	88 (4.3)	
Annual income				0.263
Below 100,000	79 (14.8)	78 (14.6)	284 (13.8)	
100,000-300,000	299 (56.1)	298 (55.9)	1,093 (53.0)	
Above 300,000	155 (29.1)	157 (29.5)	687 (33.3)	
Family history of Diabetes				0.001
Yes	24 (4.5)	15 (2.8)	36 (1.7)	
No	509 (95.5)	518 (97.2)	2,028 (98.3)	
Hypertension comorbidity				0.001
Yes	198 (37.1)	255 (47.8)	562 (27.2)	
No	335 (62.9)	278 (52.2)	1502 (72.8)	
Bedtime				0.238
Before 23:00	206 (38.6)	215 (40.3)	755 (36.6)	
After 23:00	327 (61.4)	318 (59.7)	1,309 (63.4)	
Length of sleep				0.207
<7 hours	435 (81.6)	429 (80.5)	1,650 (79.9)	
≥7 hours	98 (18.4)	104 (19.5)	414 (20.1)	
EQ-VAS^#^	79.11 ± 8.48	80.67 ± 7.72	81.65 ± 19.64	0.006
EQ-Index^#^	0.968 ± 0.054	0.972 ± 0.047	0.972 ± 0.043	0.144

PSM, propensity score matching; BMI, body mass index; EQ-VAS, EQ visual analog scale; # Data was presented as mean ± standard deviation.

The EQ-5D-5L instrument was employed as the quality of life assessment tool in this study, administered through self-completion by participants. This validated instrument consists of two components: the EQ-5D descriptive system and the EQ visual analog scale (EQ-VAS). The EQ-VAS provides a quantitative measure of self-rated health status through a 100-mm vertical scale, anchored by the descriptors “best imaginable health state” (100 points) and “worst imaginable health state” (0 points). Meanwhile, the EQ-5D-5L descriptive system evaluates health-related quality of life across five discrete dimensions: mobility, self-care, usual activities, pain/discomfort, and anxiety/depression. Responses across these dimensions were converted to an EQ-5D index value using the standardized Chinese population-based value set (26).

The investigators received comprehensive training to ensure thorough understanding of the study protocol and data collection procedures. Data entry procedures employed a dual approach: paper-based DCF utilized double data entry with discrepancy resolution, while electronic data capture systems incorporated automated validation checks. The consolidated database underwent systematic quality control measures, including comprehensive logic checks and independent verification by an independent data management team. These multi-layered validation processes were implemented to maximize data accuracy and reliability throughout the study.

### 2.7 Sample size estimation

The Sample sizes were calculated by using Gpower 3.1.2 (http://www.ats.ucla.edu/stat/gpower/) using a ANCOVA with fixed effects, considering main effects and interactions, with a desired power of 90%, at a 5% significance level, and an effect size of 0.0859. The effect size calculation derived from EQ-VAS scores (group means: 78, 80, 82) with pooled standard deviation (SD) of 19 across three experimental groups (17). The calculation indicated that a minimum of 1426 participants were required. Therefore, there were 18605 respondents in this survey, which met the requirements indicated by power analysis. Following propensity score matching (PSM), the matched sample size of 3130 still retain analytical adequacy.

### 2.8 Statistical analysis

All statistical analyses were performed using SPSS Statistics 18.0 (IBM Corporation, Chicago, IL, USA). Categorical variables were expressed as frequency percentages, while continuous variables were presented as mean ± standard deviation (SD) or median with interquartile range (IQR), based on data distribution characteristics. Missing data were systematically evaluated prior to matching and addressed through multiple imputation techniques for continuous variables and modal substitution for categorical variables. The study population was stratified into three glycemic status categories: normoglycemic (n=16,389, 88.1%), prediabetic (n=1,339, 7.2%), and diabetic (n=875, 4.7%). To address group imbalance while maintaining statistical power and maximizing analytical validity, an optimal propensity score matching (PSM) was constructed using nearest-neighbor matching (1:1:4 ratio for diabetes: prediabetes: normal glycemic group) with a caliper width of 0.01. This matching ratio was selected based on two key considerations: (1) The original sample size disparity necessitated retaining sufficient normal glycemic controls (largest group) to ensure adequate power for subgroup analyses; (2) Pilot matching tests demonstrated that 1:1:4 achieved optimal balance (standardized mean differences <0.1) while preserving 92% of diabetic cases, compared to 67% retention with 1:1:1 matching. The matching algorithm took several categorical variables as covariates, incorporated age, gender, educational attainment, body mass index (BMI), and annual income. Intergroup comparisons employed χ² tests or Fisher’s exact tests for categorical parameters, and one-way ANOVA with *post hoc* Bonferroni adjustment or Wilcoxon rank-sum tests for continuous measures, as appropriate. Multivariate linear regression models were subsequently developed, incorporating all variables demonstrating statistical significance (*P*<0.05) in univariate analyses. These models quantified the adjusted associations between glycemic status (independent variable) and HRQoL outcomes (EQ-VAS scores and EQ-Index), while controlling for identified confounders. Stratified analyses were conducted across predetermined subgroups: biological sex, age tertiles, BMI categories, and hypertension comorbidity status.

## 3 Results

### 3.1 Characteristics of subjects

**Figure 1:**
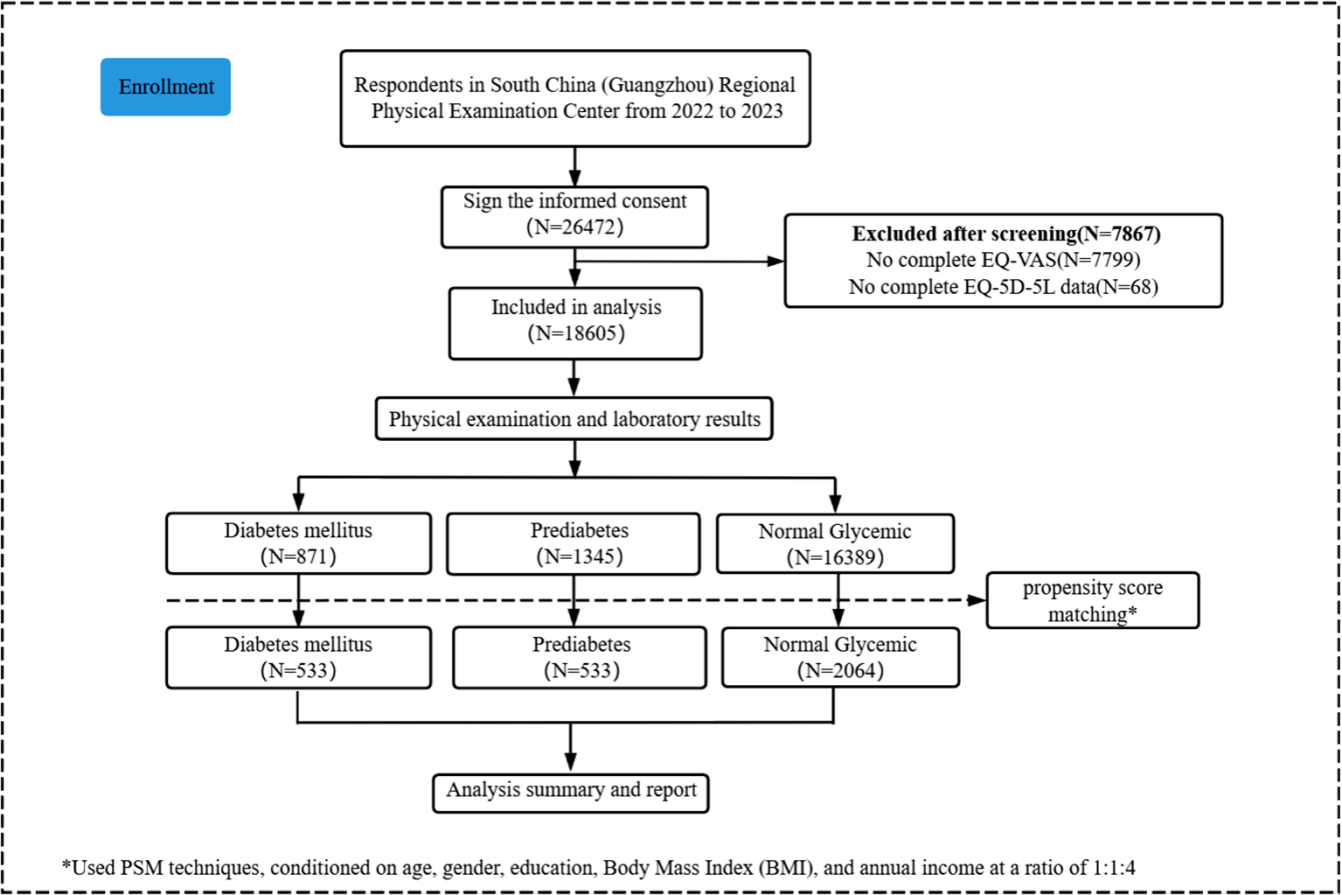
Study flowchart and participants recruited. EQ-VAS, EQ visual analog scale.

A total of 26,472 participants were enrolled, and 7,867 subjects not reporting EQ-5D-5L were excluded; then, the overall characteristics of the 18,605 subjects were outlined. **Figure 1** depicts the participant selection process through a standardized flowchart. Within the study population, diabetes mellitus prevalence was 4.7% (n=875), demonstrating significant gender disparity (male: 77.7% vs female: 22.3%). Similarly, prediabetes affected 7.2% of participants (n=1,345), with comparable gender distribution patterns (male: 74.1% vs female: 25.9%), as detailed in **Supplementary Table S1** (**Supplementary Appendix Table S1**).

Post-matching comparative analysis demonstrated no statistically significant intergroup differences (*P*>0.05) in sociodemographic parameters (age, biological sex, BMI, educational attainment, marital status, and annual income) or sleep architecture characteristics (bedtime regularity and sleep duration). However, significant disparities emerged in behavioral and clinical profiles, particularly regarding alcohol consumption patterns, familial diabetes history, and hypertension comorbidity status (*P*<0.05). **Table 1** delineates the demographic and clinical characteristics of the propensity-matched groups stratified by glycemic status (diabetes mellitus, prediabetes, and normal glycemic levels).

### 3.2 Analysis on EQ-index and EQ-VAS within groups after PS matching

Comprehensive analysis of the EQ-5D-5L descriptive system demonstrated comparable dimensional impairment patterns across propensity score-matched glycemic strata (diabetes, prediabetes, and normoglycemia). The “Pain/discomfort” and “Anxiety/depression” dimensions emerged as predominant health concerns, followed sequentially by “Mobility”, “Self-care”, and “Usual activities”. Chi-square testing revealed no statistically significant intergroup differences in dimension distribution patterns, as detailed in **Table 2** and visualized in **Figure 1**.

**Table 2:** EQ-5D-5L among the three groups after PSM.

Dimensions and levels	Diabetes mellitus (n=533)	Prediabetes (n=533)	Normal glycemic (n=2064)	*P*
	N (%)	N (%)	N (%)	
Mobility				0.090
no problems	528 (99.1)	532 (99.8)	2,058 (99.7)	
slight problems	5 (0.9)	1 (0.2)	6 (0.3)	
moderate problems	0 (0.0)	0 (0.0)	0 (0.0)	
severe problems	0 (0.0)	0 (0.0)	0 (0.0)	
extreme problems	0 (0.0)	0 (0.0)	0 (0.0)	
Self-care				0.444
no problems	531 (99.6)	533 (100)	2,058 (99.7)	
slight problems	2 (0.4)	0 (0.0)	6 (0.3)	
moderate problems	0 (0.0)	0 (0.0)	0 (0.0)	
severe problems	0 (0.0)	0 (0.0)	0 (0.0)	
extreme problems	0 (0.0)	0 (0.0)	0 (0.0)	
Usual activities				0.643
no problems	531 (99.6)	532 (99.8)	2,052 (99.4)	
slight problems	2 (0.4)	1 (0.2)	12 (0.6)	
moderate problems	0 (0.0)	0 (0.0)	0 (0.0)	
severe problems	0 (0.0)	0 (0.0)	0 (0.0)	
extreme problems	0 (0.0)	0 (0.0)	0 (0.0)	
Pain/discomfort				0.810
no problems	357 (67.0)	365 (68.5)	1,399 (67.8)	
slight problems	167 (31.3)	156 (29.3)	634 (30.7)	
moderate problems	4 (0.8)	11 (2.1)	28 (1.4)	
severe problems	5 (0.9)	1 (0.2)	3 (0.1)	
extreme problems	0 (0.0)	0 (0.0)	0 (0.0)	
Anxiety/depression				0.127
no problems	446 (83.7)	462 (86.7)	1789 (86.7)	
slight problems	77 (14.4)	65 (12.2)	256 (12.4)	
moderate problems	8 (1.5)	4 (0.8)	18 (0.9)	
severe problems	2 (0.4)	2 (0.4)	1 (0.0)	
extreme problems	0 (0.0)	0 (0.0)	0 (0.0)	

PSM, propensity score matching.

**Figure 2:**
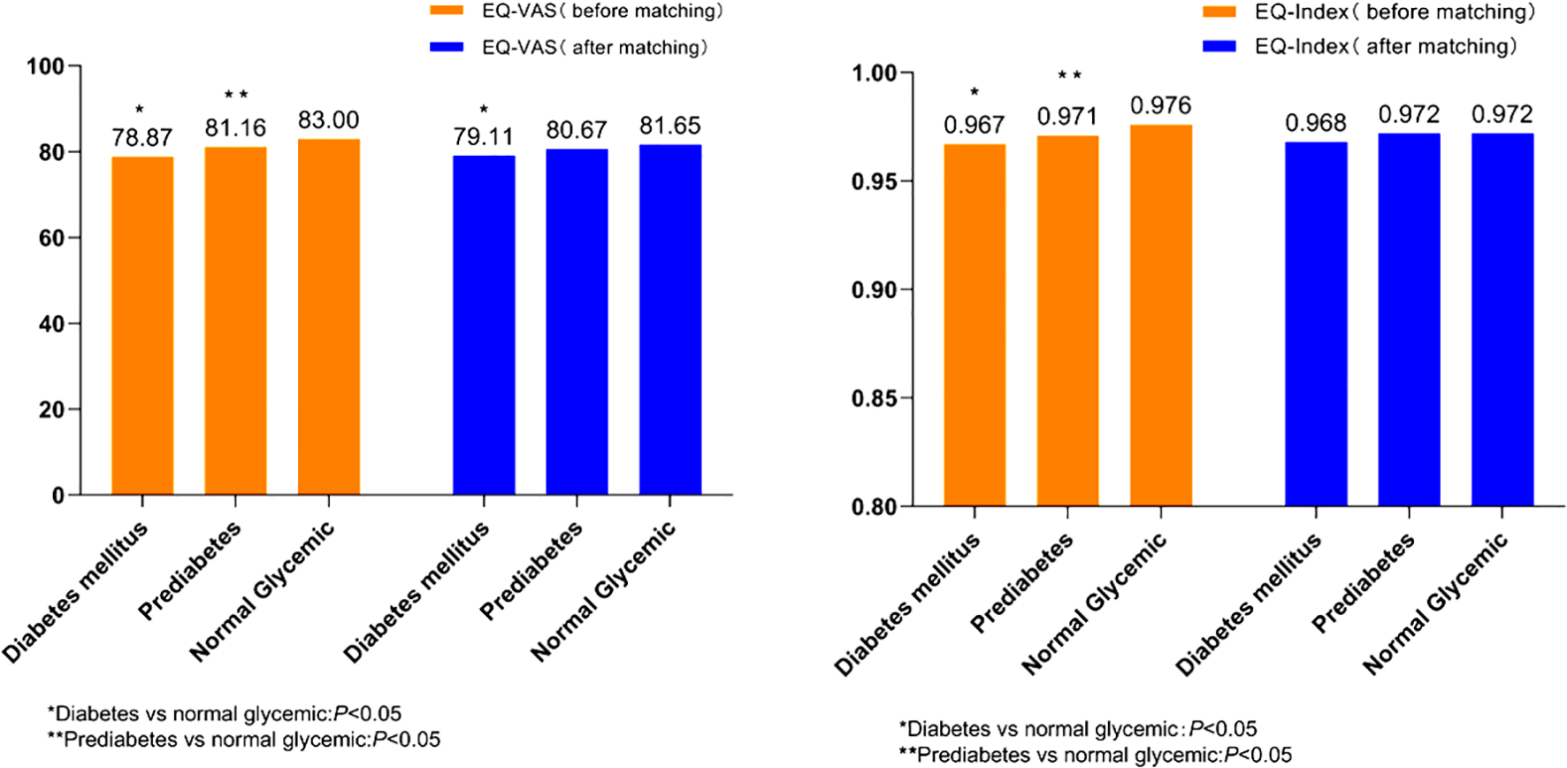
The distribution of EQ-VAS and EQ-Index in the three groups before and after propensity score matching. EQ-VAS, EQ visual analog scale. ** means that Prediabetes vs normal glycemic: P<0.05.

Consistent with these findings, composite EQ-Index scores remained not statistically differ across study groups post-matching: prediabetes (0.972 ± 0.047), diabetes mellitus (0.968 ± 0.054), and normal glycemic controls (0.972 ± 0.043) (*P*>0.05). This dimensional concordance persisted despite observed differences in raw dimension prevalence rates. Conversely, significant intergroup disparities emerged in EQ-VAS measurement. The diabetes group exhibited markedly reduced EQ-VAS scores (79.11 ± 8.48) compared to normal glycemic levels counterparts (81.65 ± 19.64) (*P*<0.05). These results are visualized in **Figure 2**.

### 3.3 Multivariate linear regression analysis on EQ-index and EQ-VAS

Multivariate linear regression models incorporating adjustments for key confounders (family history of diabetes, alcohol consumption patterns, and hypertension comorbidity status) revealed significant associations between glycemic status and HRQoL outcomes (**Table 1**). Specifically, the fully adjusted model demonstrated a 2.139-point reduction in EQ-VAS scores for the diabetes group compared to normal glycemic group (95% CI: -3.748 to -0.530; *P*=0.009) (**Table 3**, adjusted model). Conversely, no statistically significant differences in EQ-Index scores persisted after controlling for confounding variables, consistent with the preliminary intergroup comparisons (**Table 4**, adjusted model).

**Table 3:** Association of EQ-VAS scores with glycemic levels and other factors.

Variables	Unadjusted model		Multivariate model^a^	
	*B* (95% CI)	*P*	*B* (95% CI)	*P*
Diabetes vs normal	-2.536 (-4.121, -0.950)	0.002	-2.139 (-3.748, -0.530)	0.009
Prediabetes vs normal	-0.980 (-2.566, 0.605)	0.225	-0.956 (-2.554, 0.642)	0.241

a Co-variables including family history of diabetes, alcohol drinking, and hypertension comorbidity were entered into the model. EQ-VAS, EQ visual analog scale.

**Table 4:** Association of EQ-Index scores with glycemic levels and other factors.

Variables	Unadjusted model		Multivariate model^a^	
	*B* (95% CI)	*P*	*B* (95% CI)	*P*
Diabetes vs normal	-0.004 (-0.009, 0.001)	0.051	-0.003 (-0.008, 0.001)	0.163
Prediabetes vs normal	0.001 (-0.005, 0.004)	0.888	0.001 (-0.005, 0.004)	0.937

a Co-variables including family history of diabetes, alcohol drinking, and hypertension comorbidity were entered into the model.

### 3.4 Subgroup analysis on EQ-index and EQ-VAS after PS matching

**Figure 3:**
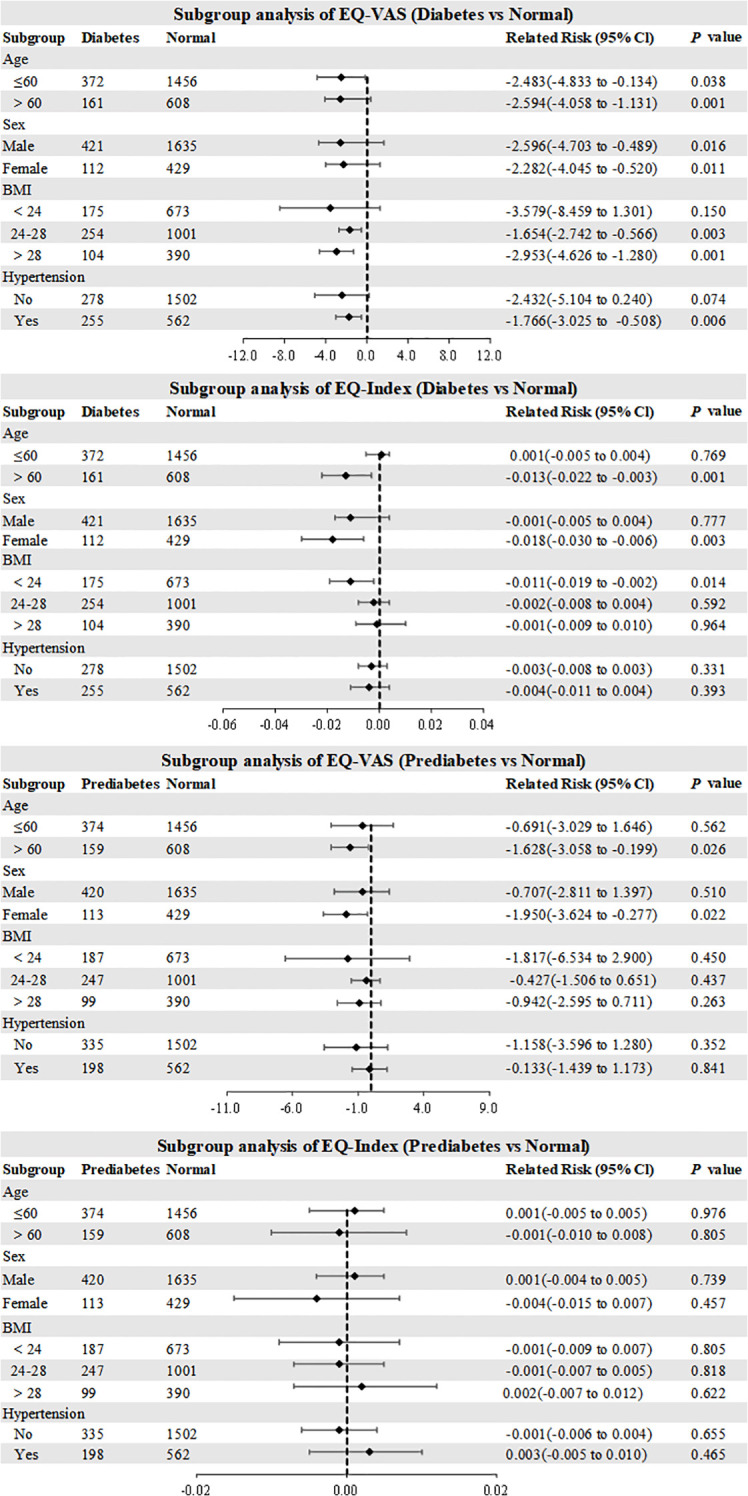
Subgroup analysis of EQ-VAS and EQ-Index. EQ-VAS, EQ visual analog scale; BMI, body mass index.

Stratified analyses revealed significant glycemic status-dependent disparities in EQ-VAS distributions across age and sex subgroups. Participants with diabetes and hypertension comorbidity demonstrated significantly reduced EQ-VAS scores (1.766-point deficit, 95% CI: -3.025 to -0.508; *P*=0.006) compared to normal glycemic levels with similar comorbidity profiles. BMI-stratified analyses showed progressive EQ-VAS reductions in diabetes subgroups: 1.654-point deficit (95% CI: -2.742 to -0.566; P=0.003) in overweight (BMI 24-28) and 2.953-point deficit (95% CI: -4.626 to -1.280; *P*=0.001) in obese (BMI≥28) categories relative to BMI-matched normal glycemic levels. EQ-Index analyses identified specific vulnerability patterns: geriatric diabetes patients (≥60 years) exhibited 0.013-point decrement (95% CI: -0.022 to -0.003; *P*=0.001) and female diabetes patients demonstrated 0.018-point reduction (95% CI: -0.030 to -0.006; *P*=0.003) compared to their respective normal glycemic subgroups (**Figure 3**).

Stratified analyses of prediabetes subgroups revealed significant EQ-VAS impairments in older adults (≥60 years: 1.628-point deficit, 95% CI: -3.058 to -0.199; *P*=0.026) compared to age-matched normal glycemic controls. Female prediabetes subjects demonstrated a 1.950-point reduction (95% CI: -3.624 to -0.277; *P*=0.022) relative to sex-matched normal glycemic levels. Notably, prediabetes status showed no statistically significant association with EQ-Index variations across any analyzed demographic or clinical subgroups (**Figure 3**).

## 4 Discussion

This population-based study provides novel evidence that impaired glucose metabolism status, including both diabetes and prediabetes, is significantly associated with reduced HRQoL as measured by the EQ-5D-5L instrument, compared with normal glycemic individuals.

Comparative analysis of HRQoL in T2DM populations reveals geographical variations in EQ-Index scores. Existing literature documents superior HRQoL profiles in Japanese research (EQ-Index=0.86) compared to Canadian (0.79) and British (0.72) populations (27, 28). Benchmarking against these multinational data, our investigation identified elevated EQ-Index values (0.968) in Guangzhou with diabetes, suggesting potential regional-specific protective factors in Southern China. Notably, dimensional analysis demonstrated exceptional functional capacity across all glycemic strata: more than 99% of participants reported unimpaired performance in “Mobility”, “Self-care”, and “Usual activities” domains. This pattern suggests pronounced ceiling effects in core functional dimensions, potentially limiting the instrument’s discriminative capacity in populations with preserved physical functioning (28–30).

EQ-5D-5L deterioration was evident in diabetes across weight categories-overweight (1.65-point EQ-VAS deficit) and obese (2.95-point EQ-VAS deficit) subgroups demonstrating amplified deficits compared to BMI-matched controls. As we all Known, the global diabetes epidemic remains inextricably linked to rising obesity prevalence (31). While BMI thresholds for clinical decision-making in T2DM management persist as contentious issues (32), our findings reveal a critical discrepancy: the observed HRQoL decline commencing at BMI≥24 kg/m² in diabetes contrasts with the National Institute for Health and Care Excellence recommended intervention threshold of 27.5 kg/m² for Chinese populations (33). This 3.5 kg/m² differential underscores clinical implications for risk stratification, suggesting potential benefits of anticipatory weight management strategies in overweight (BMI≥24 kg/m²) diabetes subgroups prior to reaching conventional intervention thresholds.

What is more, all three groups exhibited similar trends across five dimensions of EQ-5D-5L, with “Pain/discomfort” and “Anxiety/depression” emerging as the primary areas of reported problems. Compared to females of normal glycemic levels, females with diabetes or prediabetes experience more frequent and intense “Pain/discomfort” and “Anxiety/depression”; however, males do not exhibit any corresponding differences. The subgroup analysis on EQ-Index and EQ-VAS among the three groups also demonstrated the same gender differences. The synergistic relationship of female diabetes manifested as a 0.018-point EQ-Index reduction. Besides, prediabetes-associated HRQoL showed that females manifested as a 1.95-point EQ-VAS deficit demonstrating significant impairments. This might be attributed to the emotional turmoil associated with the changes in female hormones, such as endocrine and neuroendocrine (34).

Additionally, we found that elderly patients (age > 60 years) with prediabetes or diabetes got worse in HRQoL than normal glycemic levels. The synergistic relationship of geriatric (≥60 years) with diabetes exhibited compounded vulnerabilities, showing 0.013-point EQ-Index reductions respectively. Consistently, prediabetes-associated HRQoL showed that older adults manifested as 1.63-point EQ-VAS deficit demonstrating significant impairments. The following factors may contribute to the result. Firstly, elderly patients with prediabetes or diabetes are more likely to experience mobility problems caused by complications (35, 36). Secondly, the academic community found that the cognitive function of those living with diabetes is closely related to the decrease of HRQoL for the elderly (37). Further longitudinal investigations are warranted to elucidate the temporal dynamics and mechanistic pathways underlying these observed associations.

Our findings are strengthened by the inclusion of 18,605 participants, representing the largest comparative analysis of HRQoL across glycemic status groups conducted in Southern China to date. These results extend previous national findings, providing contemporary epidemiological evidence related to quality of life in three glycemic subgroups (diabetes, prediabetes, and normoglycemia) in Southern China for health policy formulation. This study also has several limitations that may lead to specific biases. Single-center and the recruitment through a hospital-based health management center design may introduce selection bias, limiting generalizability to populations with differing healthcare access or regional lifestyles. Cross-sectional methodology precludes temporal assessment of glycemic progression and HRQoL dynamics, potentially obscuring causal relationships. Thus, we plan to conduct a large-sample prospective cohort study to explore the more profound development and cross-correlations of HRQoL development and cross-correlations among individuals with diabetes, prediabetes, and normal glycemic levels. The crucial aspect for the future is how aged 60 and above, female, overweight or obese (BMI > 24kg/m^2^) impact the HRQoL in the populations with diabetes, prediabetes, and normal glycemic levels, and it needs to be explored in the future longitudinal studies.

## 5 Conclusions

Prediabetes and diabetes are linked to lower HRQoL than normal glycemic levels. Individuals with hypertension comorbidity, aged 60 and above, female, overweight or obese (BMI > 24kg/m^2^) are found to be correlated with decreased HRQoL in prediabetes and diabetes, which need more care to enhance their HRQoL to live normal lives, aligning with the objectives of diabetes management strategies.

## Data Availability

The datasets presented in this study can be found in online repositories. The names of the repository/repositories and accession number(s) can be found in the article/**Supplementary Material**.
