# Supplementary material for: Health-related quality of life in populations with diabetes, prediabetes, and normal glycemic levels in Guangzhou, China: a cross-sectional study
Source: Front Endocrinol (Lausanne). 2025 May 23;16:1518204. doi: 10.3389/fendo.2025.1518204 (PMC12140990; doi:10.3389/fendo.2025.1518204)
Supplement: Supplementary file 1 [file Table1.docx]

**Supplementary Appendix**

**Supplementary Appendix Table 1 General characteristics (before propensity score matching)**

| **Characteristic** | **Diabetes mellitus (n=871)** | **Prediabetes (n=1345)** | **Normal Glycemic (n=16389)** | ***P*** |
| --- | --- | --- | --- | --- |
|  | N (%)/(MD±SD) | N (%)/(MD±SD) | N (%)/(MD±SD) |  |
| **Sex**  Male  Female | 677(77.7)  194(22.3) | 997(74.1)  348(25.9) | 10462 (63.8)  5927(36.2) | 0.001 |
| **Age** | 58.77±10.35 | 54.15±11.37 | 43.59±11.62 | 0.001 |
| **BMI** | 25.20±3.31 | 25.96±3.33 | 23.86±3.15 | 0.001 |
| **Cigarette smoking**  Non-smoker  Former smoker  ≤20 year  ＞20 year | 577(66.2)  97(11.1)  71(8.2)  126(14.5) | 959(71.3)  129(9.6)  113(8.4)  144(10.7) | 12978(79.2)  871(5.3)  1590(9.7)  950(5.8) | 0.001 |
| **Alcohol drinking**  Non-Alcohol  Former drinker  <5 times/week  ≥ 5 times/week | 430(49.4)  37(4.2)  388(44.5)  16(1.8) | 441(32.8)  38(2.8)  843(62.7)  23(1.7) | 7935(48.4)  240(1.5)  8114(49.5)  100(0.6) | 0.001 |
| **Education levels**  ≤Bachelor degree  >Bachelor degree | 145(16.6)  726(83.4) | 138(10.3)  1207(89.7) | 748(4.6)  15641(95.4) | 0.001 |
| **Marital status**  Unmarry  Divorce  Widow  Marry  Remarry | 21(2.4)  31(3.6)  26(3.0)  755(86.7)  38(4.4) | 35(2.6)  64(4.8)  33(2.5)  1162(86.4)  51(3.8) | 2367(14.4)  646(3.9)  120(0.7)  12799(78.1)  457(2.8) | 0.001 |
| **Annual income**  Below 100,000  100,000-300,000  Above 300,000 | 137(15.7)  495(56.8)  239(27.4) | 172(12.8)  716(53.2)  457(34.0) | 1347(8.2)  8671(52.9)  6371(38.9) | 0.001 |
| **Family history**  Yes  No | 838(96.2)  33(3.8) | 1293(96.1)  52(3.9) | 16074(98.1)  315(1.9) | 0.001 |
| **Bedtime**  Before 23:00  After 23:00 | 345(39.6)  526(60.4) | 497(37.0)  848(63.0) | 4368(26.7)  12021(73.3) | 0.001 |
| **Length of sleep**  <7 hours  ≥7 hours | 703(80.7)  168(19.3) | 1074(79.9)  271(21.1) | 12426(75.8)  3963(24.2) | 0.001 |
| **EQ-VAS** | 78.87±8.43 | 81.16±22.65 | 83.00±18.65 | 0.001 |
| **EQ-Index** | 0.967±0.055 | 0.971±0.046 | 0.976±0.041 | 0.001 |

EQ-VAS: EQ visual analog scale; BMI:body mass index.
