# Supplementary material for: Health-related quality of life in populations with diabetes, prediabetes, and normal glycemic levels in Guangzhou, China: a cross-sectional study
Source: Front Endocrinol (Lausanne). 2025 May 23;16:1518204. doi: 10.3389/fendo.2025.1518204 (PMC12140990; doi:10.3389/fendo.2025.1518204)
Supplement: Supplementary file 2 [file Table2.docx]

**Supplementary Appendix Appendix Table 2 EQ-5D-5L by the three groups (before propensity score matching)**

| **Dimensions and levels** | **Diabetes mellitus (n=871)** | **Prediabetes (n=1345)** | **Normal Glycemic (n=16389)** |
| --- | --- | --- | --- |
|  | N (%) | N (%) | N (%) |
| **Mobility**  no problems  slight problems  moderate problems  severe problems  extreme problems | 858(98.5)  11(1.3)  2(0.2)  0 (0.0)  0 (0.0) | 1338(99.5%)  5(0.4)  2(0.1)  0 (0.0)  0 (0.0) | 16364(99.8%)  21(0.1)  3(0.0)  1 (0.1)  0(0.0) |
| **Self-care**  no problems  slight problems  moderate problems  severe problems  extreme problems | 863(99.1)  8(0.9)  0 (0.0)  0 (0.0)  0 (0.0) | 1345(100.0)  0 (0.0)  0 (0.0)  0 (0.0)  0 (0.0) | 16372(99.9)  13(0.1)  3(0.0)  0(0.0)  1(0.0) |
| **Usual activities**  no problems  slight problems  moderate problems  severe problems  extreme problems | 859(98.7)  10 (1.1)  2 (0.2)  0 (0.0)  0 (0.0) | 1339(99.6)  6(0.4)  0(0.0)  0 (0.0)  0 (0.0) | 16357(99.8)  28(0.2)  2(0.0)  2(0.0)  0(0.0) |
| **Pain/discomfort**  no problems  slight problems  moderate problems  severe problems  extreme problems | 566(65.0)  291(33.4)  9(1.0)  5(0.6)  0(0.0) | 924(68.7)  399(29.7)  21(1.6)  1(0.1)  0(0.0) | 12197(74.4)  4026(24.6)  151(0.9)  15(0.1)  0(0.0) |
| **Anxiety/depression**  no problems  slight problems  moderate problems  severe problems  extreme problems | 744(85.4)  114(13.1)  10(1.1)  3(0.3)  0(0.0) | 1141(84.8)  189(14.1)  12(0.9)  2(0.1)  1(0.1) | 13950(85.1)  2323(14.2)  106(0.6)  10(0.1)  3(0.0) |
