# Supplementary figures and images for: Health-related quality of life in populations with diabetes, prediabetes, and normal glycemic levels in Guangzhou, China: a cross-sectional study
Source: Front Endocrinol (Lausanne). 2025 May 23;16:1518204. doi: 10.3389/fendo.2025.1518204 (PMC12140990; doi:10.3389/fendo.2025.1518204)

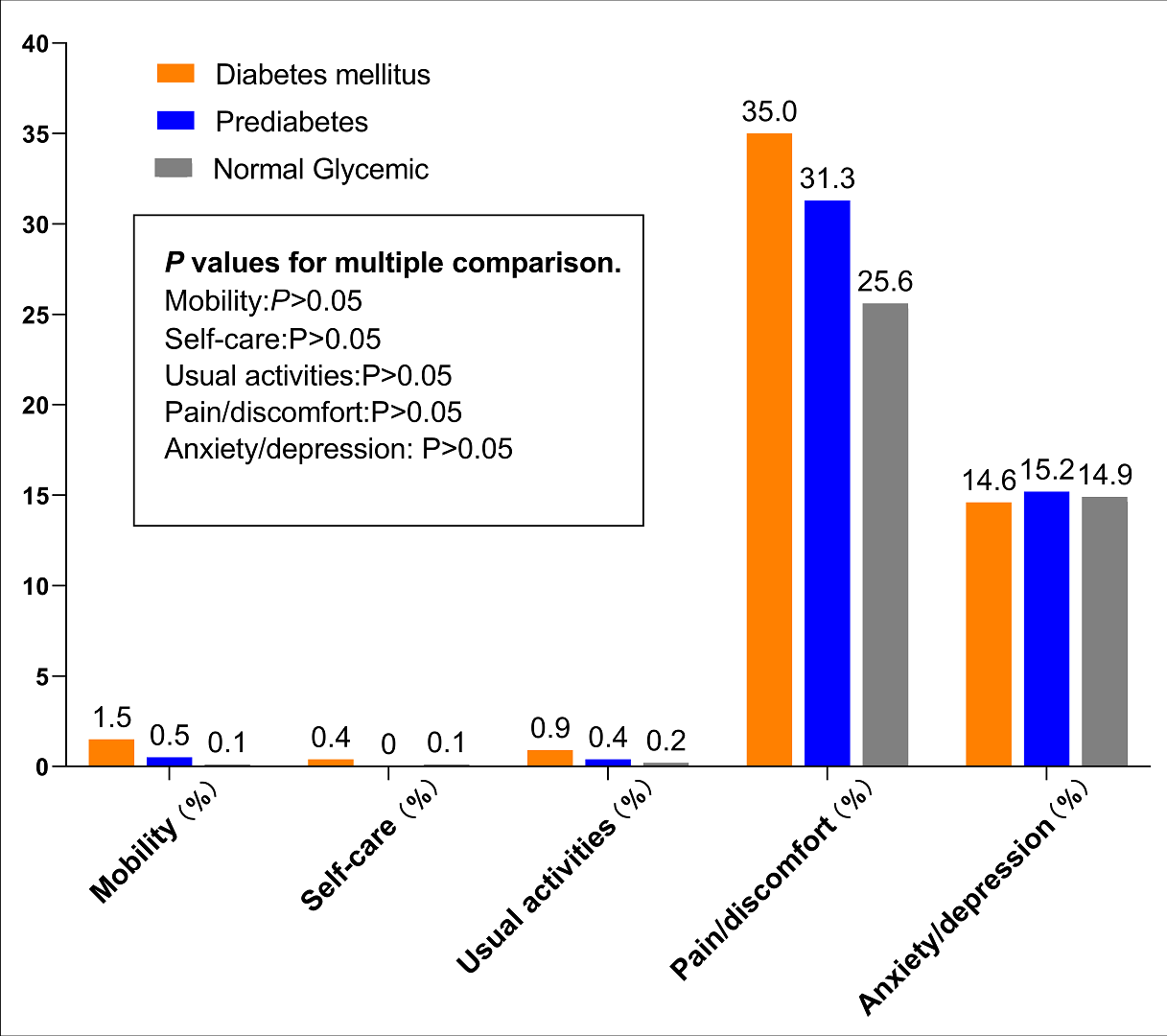

Supplement: Supplementary file 3 [file Image1.tif]
